# Effectiveness of zinc chloride mouthwashes on oral mucositis and weight of patients with cancer undergoing chemotherapy

**DOI:** 10.1186/s12903-021-01706-w

**Published:** 2021-07-22

**Authors:** Khodayar Oshvandi, Seyed Yaser Vafaei, Seyed Ramesh Kamallan, Salman Khazaei, Hossein Ranjbar, Fateme Mohammadi

**Affiliations:** 1 Department of Medical Surgical Nursing, School of Nursing and Midwifery, Mother and Child Care Research Center, Hamadan University of Medical Sciences, Hamadan, Iran; 2 Department of Pharmaceutics & Pharmaceutical Biotechnology, School of Pharmacy, Hamadan University of Medical Sciences, Hamadan, Iran; 3 Department of Medical Surgical Nursing, Student Research Center, Hamadan University of Medical Sciences, Hamadan, Iran; 4 Department of Epidemiology, Health Sciences Research Center, Health Sciences and Technology Research Institute, Hamadan University of Medical Sciences, Hamadan, Iran; 5 Department of Hematology Oncology, Department of Internal Medicine, School of Medicine, Shahid Beheshti Medical Educational Center, Hamadan University of Medical Sciences, Hamadan, Iran; 6 Department of Pediatric Nursing, Chronic Diseases (Home Care) Research Center and Autism Spectrum Disorders Research Center, Hamadan University of Medical Sciences, Hamadan, Iran

**Keywords:** Zinc chloride, Prevention, Oral mucositis, Chemotherapy

## Abstract

**Background:**

Oral mucositis is one of the most emerging and debilitating complications of chemotherapy during the treatment period, which strongly affects the nutritional status and physical and mental condition of these patients. Zinc increased protein synthesis and improved cell membrane stability so passible effective in prevent and treat oral mucositis and promote oral health. Therefore, this study aimed to evaluate the effect of zinc chloride mouthwash on the prevention, incidence, and severity of oral mucositis in cancer patients undergoing chemotherapy.

**Methods:**

The present study was a randomized control trial study. 96 patients with a cancer diagnosis selected from one oncology clinic in the west of Iran. Then they assigned randomly to the zinc chloride group and placebo group. The patients in each group should rinse their mouths every 8 h two times and each time 2 min with 7.5 ml from mouthwash. The severity of mucositis and weight loss examined blindly at the baseline and 3-week follow-up.

**Results:**

The incidence and severity of oral mucositis between groups were significant higher at the end of the second (p < 0.002) and third (p < 0.001) week. The mucositis severity decreased well during the third weeks in the zinc chloride group. The difference in the weight loss was significant higher between the zinc chloride and the placebo group (p < 0.01).

**Conclusion:**

Zinc chloride mouthwash was effective in preventing and reducing the severity of oral mucositis and improving weight in patients undergoing chemotherapy.

*Trial registration* We can therefore recommend more studies examine the effects zinc chloride as preventive care at the beginning of chemotherapy to improve oral health and subsequently preventing weight loss in these patients.

## Background

Cancer is one of the most horrible diseases of the century because it has affected more than 15 million people worldwide [1]. And, it has caused more than 7 million deaths annually in the world [2]. Studies conducted in Iran show that over 112,000 people are diagnosed with breast, colorectal, prostate, stomach, and blood cancers, of which 54% are male and 46% of patients are female [3]. The growing statistics of cancer in Iran caused the center for disease control and prevention to implement strategies with an emphasis on the three goals of prevention, early diagnosis, and effective treatment with the least side effects [4]. In this regard, one of the most important strategies is to provide effective treatment with the least side effects of care therapy, which emphasizes the importance of paying attention to choosing the appropriate treatment and providing efficient care by medical staff [4, 5]. While chemotherapy is one of the most feasible treatments for patients with various types of cancer [6] but this treatment method causes many problems and side effects for the patient [7]. The most significant and common side effects of chemotherapy are nausea, vomiting, hair loss, loss of appetite, impaired body image and Oral mucositis [8, 9]. Oral mucositis is severe, painful inflammation with multiple lesions of the oral. Mucositis appear from the fourth day of chemotherapy in 40 to 80% of patients undergoing chemotherapy that usually lasts 7 to 14 days after chemotherapy and causes a lot of pain and discomfort in these patients [10–12]. Because these patients experience persistent and excruciating pain in the oral cavity when eating and even drinking fluids and talking, which severely affects their nutritional status, physical condition [5, 10], Consequently, their mental health and quality of life [2, 5, 10]. However, despite the high incidence of oral mucositis in patients undergoing chemotherapy, there is no available definitive treatment for mucositis, and only supportive cures were used to reduce pain associated with mucositis [13].

The multinational association of supportive care in cancer (MASCC) has provided a guideline and appropriate recommendations for the prevention and treatment of oral mucositis in cancer patients. This panel suggests that implementation of multi agent combination oral a care protocol is beneficial for the prevention of oral mucositis during chemotherapy [14]. Thus, this has led to several studies in recent years trying to find a way to reduce the incidence and treatment of oral mucositis in these patients [5, 10, 15]. Yarom et al. [16] stated zinc supplements orally effective in the prevention of oral mucositis in oral cancer patients receiving radiation therapy or chemo radiation. In this regard, some studies have investigated the effectiveness of zinc on oral mucositis in these patients, because zinc a vital role in promoting regeneration processes, cell membrane stability, and membrane wound healing by increasing protein synthesis and nucleic acid as well as improving oxygen transfer [10–12]. Although high consumption of zinc in some people might cause nausea, vomiting, diarrhea, metallic taste, kidney and stomach damage, and other side effects, but zinc is safe when taking by mouth in doses 40 mg daily [5]. Therefore, studies have used the safe dose of zinc to evaluate the effect of zinc on the prevention and treatment of oral mucositis in patients with cancer [5, 10, 12]. These studies have shown the effectiveness of zinc sulfate capsules in the prevention and treatment of oral mucositis in cancer patients undergoing chemotherapy [5, 12]. Unfortunately, in these circumstances, zinc is mostly used in the form of zinc sulfate to treat oral mucositis, and due to severe nausea, patients undergoing chemotherapy are often reluctant to take zinc sulfate capsules and prefer to use mouthwashes over oral medications [15]. In addition, there is no care to prevent oral mucositis in patients undergoing chemotherapy, and if oral mucositis develops in these patients, the doctor prescribes one of the mouthwash brands available in the market for them. However, in many cases, the financial condition of the cancer patient is not good, these conditions become more difficult and sometimes it is difficult to provide mouthwash, which is often expensive, for patients undergoing chemotherapy. Therefore, the researchers decided to study the effect of zinc chloride mouthwash on the prevention of incidence and severity of oral mucositis in cancer patients undergoing chemotherapy to achieve acceptable results to introduce an appropriate mouthwash to prevent and treat oral mucositis in these patients.

## Method

### Design

The present study was a randomized controlled trial with one intervention and a control group conducted in a double-blind manner.

### Sample and setting

The study conducted in one oncology clinic affiliated to the University of Medical Sciences in western Iran from September 2019 to August 2020. Design of study was approved by the ethics committee of the Hamadan university of medical Sciences (Umsha.rec.1398.335), and recorded at the clinical trials Ccentre (IRCT 20160110025929N28). The written informed consent obtained from all the participants after providing them with sufficient information on the study. The sample size for this study calculated based on Chou et al. study with power of 80% and α = 0.05 by using formula [17]. therefore, about 44 patients was estimated for each group, which with the loss of about 10% of the samples during the study, sample size was finally considered 48 people in each group.
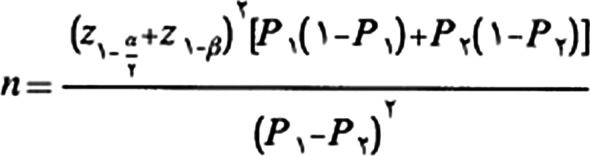


Inclusion criteria included: patients with stage 1 or 2 cancer, undergoing chemotherapy, age above 18 years, lack of oral mucositis, being alert and able to use mouthwash unaided, receiving same chemotherapy (fluorouracil, mitoxantrone), lack of leukemia, lack of pregnancy, lack of breast feeding, lack of history of smoking, lack of immune deficiency, lack of oral cavity disease prior to the start of chemotherapy, lack of underlying diseases (diabetes as well as liver, renal and digestive diseases) and willingness to participate in the study. Exclusion criteria were thrombocytopenia, worsening of the patient's condition, increase in the degree of oral mucositis and treatment discontinue for any reason.

### Recruitment and allocation

After determining the sample size, a total of 106 patients were screened for eligibility; 10 patients were ineligible and finally, 96 patients who gave written informed consent were enrolled and randomly assigned to one of two groups (zinc chloride mouthwash, 48 subjects and control, placebomouthwas,48 subjects) using block randomization. Sequentially numbered, opaque, sealed bottles with similar shape, color and size containing of zinc chloride mouthwash and placebo were used to conceal the allocation sequence and to maintain blinding and necessary measures were taken so that the sequence would remain concealed until the intervention was allocated to the intervention groups. The allocation sequence and packages were prepared by a pharmacologist, a faculty member of Hamadan university of medical Sciences, did not involve in the recruitment, data collection and analysis.

### Procedures

The protocol of this study was designed based on the opinion of a pharmacologist and trusting the protocol of two studies Cabrera et al. and Rambod et al. [5, 15]. Therefore, mouthwashes for all patients of zinc chloride and placebo in this study were prepared by a pharmacist specializing in pharmaceutics in the clean room of Hamadan university of medical sciences. Mouthwash for the zinc chloride group containing zinc chloride 0.2%, greasy mint, preservative and mouthwash for the control group were similar to the intervention group but lacked effective material. All patients in two groups were educated in routine oral care (teeth brushing, proper nutrition, and mouth hygiene). The patients in each group should rinse their mouths every 8 h two times and each time 2 min with 7.5 ml from mouthwash. The time interval between each mouthwash was 15 min. Participants in the two groups rinsed their mouth so that the mouthwash covered the tongue, palate, throat, inside the cheeks, and all the tissues of the mouth well.

### Measures

#### Demographic information

These included age, sex, marital status, employment and finances, type of cancer, duration of cancer, frequency of chemotherapy, number of decayed teeth, and history of oral and gingival disease.

#### Outcome measures

The severity of oral mucositis was measured by using the world health organization criteria for grading of oral mucositis. This scale measures objective (redness, edema, and wound development) and subjective symptoms (dysphagia and mucosal severity). Accordingly, mucositis is classified to five levels (0–4). Asymptomatic (0); ulcers, redness in the mucous membranes of the mouth, gums, tongue, and palate is low (1); there are redness, inflammation, and multiple ulcers in the patient’s mouth, but they can eat solid food without pain (2); significant redness, inflammation and ulcers in the mouth and the ability to eat soft foods and liquids (3). Redness, inflammation and several ulcers in the mouth with pain and difficulty swallowing that make it impossible to eat food (4). This scale has content and face validity and internal reliability of 0.91 in Iranian society [18, 19]. The clinical nurse in oncology clinic with ten years of experience(assistant researcher) who was not aware of the allocation of individuals in intervention and control groups observed patients' mouths and completed the world health organization criteria in the baseline, first, second and third weeks for each patient and recorded mucositis grade.

#### Weight of the patients

Weight of the patients assessed at two time points: baseline and 3 week after the intervention at a specific time (7 am). Patients wore the same clothes each time that weight was measured using a digital scale (Beurer®, Germany). This digital scale was calibrated each week by a medical engineer. The clinical nurse in oncology clinic with ten years of experience(assistant researcher) who was not aware of the allocation of individuals in intervention and control groups observed and reported patients' weight the at the beginning and end of the study (third week).

### Data analysis

Data analyses were conducted using SPSS version 22. To analyze the data, descriptive statistics (namely frequency, percentage, mean, standard deviation) was used and inferential statistics including chi square and fisher’s exact test (comparison of data distribution), covariance (adjustment of confounding variables), Mann Whitney U test (comparison oral mucositis between groups in non-normal distribution), Friedman’s nonparametric test (comparison oral mucositis intra-group comparison). The independent t test (comparison of weight of the patients between groups) and dependent t test comparison of weight of the patients (intragroup comparison). P values of less than 0.05 were considered statistically significant for all tests expect of Mann Whitney U test that p values of less than 0.00025 were considered statistically significant.

## Results

### Sample characteristics

The average age of patients participating in this study was 46.21 ± 2.42 years in the zinc chloride group and 46.22 ± 2.33 years in the placebo. Most of the patients in two groups were male, married, and had diploma education. Most had an average income of 500 Dollars. The results showed that there was no statistically significant difference between the two groups in terms of demographic information (Table 1).

**Table 1:** Demographic information of this study participants

Demographic variables		Zinc chloride group N (%)	Placebo group N (%)	χ^2^ p value
Age	23–33	9 (18.75)	6 (12.50)	χ^2^ = 2.71, p = . 912*
	34–44	15 (31.25)	14 (29.17)	
	45–55	19 (39.58)	19 (39.58)	
	56–66	5 (10.41)	9 (18.75)	
Gender	Female	23 (47.91)	22 (45.84)	χ^2^ = 3.31, p = 901*
	Male	25 (52.09)	26 (54.16)	
Marital status	Single	13 (27.09)	11 (22.91)	χ^2^ = 3.89, p = .823*
	Married	35 (72.91)	77.09 (37)	
Education	Illiterate	5 (10.41)	5 (10.41)	χ^2^ = 3.21, p = .863**
	Primary	7 (14.58)	6 (12.50)	
	Diploma	26 (54.16)	28 (58.33)	
	Bachelor	5 (10.41)	6 (12.50)	
	Master degree and higher	5 (10.41)	3 (6.25)	
Cancer type	Liver	7 (14.58)	9 (18.75)	χ^2^ = 1.34, p = .832**
	Stomach	6 (12.50)	8 (16.67)	
	Colon	6 (12.50)	5 (10.41)	
	Uterus	6 (12.50)	5 (10.41)	
	Breast	4 (8.34)	4 (8.34)	
	Kidney	7 (14.58)	6 (12.50)	
	Bladder	5 (10.41)	4 (8.34)	
	Lung	7 (14.58)	6 (12.50)	
Job	Self-employed	13 (27.09)	15 (31/25)	χ^2^ = 1.91, p = .862*
	Employee	14 (29.17)	13 (27.09)	
	Livestock and Farmer	14 (29.17)	15 (31.25)	
	Housewife	7 (14.58)	5 (10.41)	
Number of decayed teeth	0	39 (81.25)	42 (87.50)	χ^2^ = 2.53, p = . 634**
	1	3 (6.25)	2 (4.16)	
	2	2 (4.16)	1 (2.08)	
	3	3 (6.25)	2 (4.16)	
	More than 3	1 (2.08)	1 (2.08)	
History of oral and gingival disease	Yes	5 (10.41)	3 (6.25)	χ^2^ = 1.74, p = .761**
	No	43 (89.59)	45 (93.75)	
Chemotherapy regimens	Fluorouracil	28 (58.34)	30 (62.50)	χ^2^ = 1.14, p = .812*
	Mitoxantrone	20 (41.66)	18 (37.50)	
Weight	Mean (SD)	67.89 (1.17)	68.24 (1.32)	T = 2.53, p = .0.97***

Values are expressed as no. (%)

*Chi-square test

**Fisher exact test

***Independent T test

### Oral mucositis severity

**Fig. 1:**
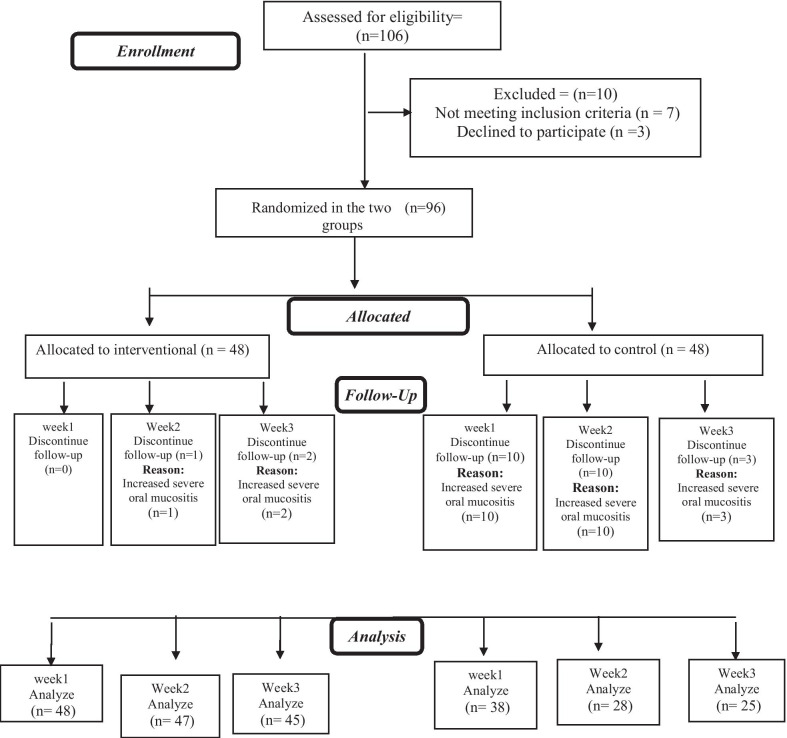
The study design

106 patients with cancer were present in the oncology clinic, 10 of whom had not the inclusion criteria, so 96 patients arrived to the study) 48 patients in each group). However, 45 patients in the zinc chloride group, and 25 patients in the placebo group completed this study. 23 patients in the placebo group left the study due to the increased severe oral mucositis, 10 patients during the first week, 7 patients during the second week and 3 patients during the third week with grade 3 oral mucositis, and 3 patients left study with grade 2 oral mucositis during the second week. 3 patients in the zinc chloride group left the study due to the increased severe oral mucositis during the second and third week (Fig. 1).

The prevalence of grades of oral mucositis between groups was significant at the end of the first (p < 0.046), second (p < 0.01) and third (p < 0.01) weeks. Accordingly, there was not oral mucositis with 1, 2 and 3 grades in the zinc chloride group end of in the first week. However, there was one patient with the grade 2 oral mucositis in the zinc chloride group in the second week and two patients with the oral mucositis with 3 grade in third week (Table 2).

**Table 2:** Oral mucositis grades between groups during this study

Mucositis grade	Zinc chloride group N (%)	Placebo group N (%)	p value
*Week 1*			
Grade 1	0 (0)	0 (0)	0.046*
Grade 2	0 (0)	0 (0)	
Grade 3	0 (0)	10 (20.83)	
*Week 2*			
Grade 1	0 (0)	0 (0)	0.01*
Grade 2	1 (2.08)	3 (10.34)	
Grade 3	0 (0)	7 (18.42)	
*Week 3*			
Grade 1	0 (0)	0 (0)	0.01*
Grade 2	0 (0)	0 (0)	
Grade 3	2 (4.16)	3 (10.34)	

Values are expressed as no. (%)

*Fisher exact test

In addition, the results of the Freidman test showed that the effect of time on severity of oral mucositis was not significant in the zinc chloride group (p = 0.41), accordingly, the severity of mucositis in the zinc chloride group controlled from the first week until third week. To compare the study groups at different time intervals, the results of the Mann Whitney U test showed that there was significant difference between the groups at the end of the first second (p = 0.002) and the third weeks (p < 0.001). This finding indicates that zinc chloride mouthwash appropriately prevented of oral mucositis from the beginning of the study until the third week compared to placebo (Table 3). However, in this study, zinc chloride mouthwash was able to reduce the incidence of oral mucositis (93.75%).

**Table 3:** Comparison of oral mucositis at different time points between groups

Groups	1 week after intervention Mean rank	2 weeks after intervention Mean rank	3 weeks after intervention Mean rank	p value
Zinc chloride group	0.02	0.52	1.01	0.41*
placebo group	0.42	2.76	3.57	0.027*
p value	0.026**	0.002**	0.001**	

*Friedman’s test was used for comparison within groups (significant p < 0.05)

**Mann–Whitney U test was used for comparison between groups (significant p < 0.0025)

### Weights of the patients

Comparison of weights of the patients between groups with using the independent t test showed no significant difference between the groups at the beginning of the study (p > 0.05), But at the end of the third week, there was significant difference between the zinc chloride group with placebo group (p < 0.01). In addition, the results of the dependent t test showed that the effect of time on weights of the patients was significant for the zinc chloride group (p = 0.039). (Table 4).

**Table 4:** Comparison of weight at different time points among groups

Groups	Baseline Mean	3 weeks after intervention Mean(SD)	p value
Zinc chloride group	67.89 (1.17)	71.61 (1.21)	0.039*
placebo group	68.24 (1.32)	67.18 (1.14)	0.181*

*Paired t test (significant p < 0.05)

## Discussion

The results of the present study have revealed that zinc chloride mouthwashes are effective in preventing and treating oral mucositis during third week. In effect, 45 patients in the zinc chloride group and 25 patients in the placebo group did not show symptoms of oral mucositis within third week of starting the study. Additionally, the findings of this study showed that there was a statistically difference between the zinc chloride group with the placebo group in prevent and reduce the severity of oral mucositis. In addition, only three patients in the zinc chloride group with grade 2 and 3 oral mucositis did not improve within third week of follow-up. Based on this, It seems that zinc chloride mouthwash is effective in prevent oral mucositis and reducing the severity of oral mucosal in cancer patients undergoing chemotherapy.

In this regard, Elad et al. Stated the provision of medicinal compounds is essential for the prevention and treatment of oral mucositis in patients with cancer undergoing chemotherapy [14]. In addition, although Yarom et al.'s study showed oral zinc compounds have been effective in the prevention and treatment of oral mucositis in patients with oral cancer [16], but the effect of zinc sulfate mouthwash has not been studied on oral mucosa in these patients.

Consistent with the findings of this study, Rambod et al.'s study also states that zinc sulfate capsules also partially prevent the development of oral mucositis in patients with leukemia undergoing chemotherapy up to 60% [5]. However, in this study, zinc chloride mouthwash reduced the incidence of oral mucositis much more. The possible reason for this difference could be the administration of zinc chloride mouthwash instead of zinc sulfate capsule and intervention only in patients with leukemia. In the present study, patients were followed up for 3 week from the start of chemotherapy, while Rambod et al. did the Follow up for patients in 14 days [5]. And because the risk of oral mucositis in patients undergoing chemotherapy increases over time, and since it can occur up to 21 days after chemotherapy, follow-up of patients up to 3 week and fewer incidences of oral mucositis in them compared to Rambod study may be due to using zinc glyceride mouthwash solution instead of zinc sulfate capsule. That is because most of these patients suffer from nausea, vomiting, and pain in swallowing the capsule is but using mouthwash is easier for them. Consistent with the findings of this study, other studies, despite using zinc sulfate capsules in the prevention of oral mucositis, they have shown that zinc can be efficient in preventing oral mucositis. In this regard, Ertekin et al. (2004) stated that zinc sulfate efficiently prevents oral mucositis in patients with head and neck cancer undergoing radiotherapy as it occurred in only 13 patients in the zinc sulfate pharyngeal mucosa group (grade 0–1–2) [12]. In the present study, the incidence of oral mucositis in cancer patients undergoing chemotherapy was three. This difference is probably due to differences in the type of cancer, treatment methods, and instruments in measuring oral mucositis. On the other hand, the findings of this study showed that zinc chloride mouthwash solution significantly reduces the severity of oral mucositis compared to the placebo group during three week. Consistent with the findings of this study, other studies have shown that zinc sulfate capsules significantly reduce the severity of oral mucositis in cancer patients undergoing chemotherapy and radiotherapy [5, 10, 12, 20–22].

In this regard, Rambod et al. state that the severity of oral mucositis in the zinc sulfate group is less mild, and there is a statistically significant difference with the placebo group [5]. Mehdipour et al. also state that the severity of oral mucosal pharynx in patients undergoing radiotherapy in the zinc sulfate group is less than placebo [10]. Other studies have shown that there is a statistically significant difference between the placebo group and the zinc sulfate group in terms of severity of oral mucositis [21, 22]. Because zinc plays a vital role in promoting the body's physiological processes, including growth and development, immune system survival, cell membrane stability, and membrane wound healing by increasing protein synthesis and nucleic acid, as well as improving oxygen transfer [23]. Therefore, in the prevention and treatment of oral mucositis in cancer patients under treatment, it can prevent tissue damage, including oral mucositis, by promoting the activity of immune cells, and even improve the repair process of tissue damage by increasing protein and nucleic acid synthesis [22]. Therefore, the administration of drugs containing zinc, especially mouthwashes, due to easier administration, it can remarkably reduce the incidence and severity of oral mucositis in cancer patients undergoing chemotherapy.

In this study also, weight loss in patients in the intervention group was prevented well during 3 weeks. Even the weight of patients in the zinc chloride group improved than placebo group. Although, some studies have examined the weight of patients with cancer, but few studies have examined the effect of herbal and chemical drugs on the oral mucositis and consequently its effect on weight in these patients. In this regard, the study of Toyomasu et al. showed that an amino acid diet is effective in treating oral mucositis and consequently is caused the body weight loss in the this group was smaller than that in the control group [24]. Also, Pathak et al. stated that significant weight loss during the treatment was seen in all patients of the control compared to the patients in the glutamine [25]. Arezoo Khanjani pour-fard-pachekenari et al. presented the difference in the weight was significant between the honey mouthwash and the control groups at the end of the third week [19].

### Limitations of the study

One of the most significant limitations of the present study was the small sample size of the participants. Therefore, it is suggested that similar studies with greater sample size be conducted in the coming years to estimate the effects of chloride mouthwash on the incidence and severity of oral mucositis more precisely and cause oral health in cancer patients undergoing chemotherapy subsequently. In addition, only patients with cancer undergoing chemotherapy were studied in this study; therefore, due to the high nausea of ​​these patients, swallowing problems, and easier administration of mouthwash than a capsule, it is suggested to investigate the effect of zinc chloride mouthwash on the prevention and treatment of oral mucositis in cancer patients undergoing radiotherapy. Another limitation was evaluating only oral mucositis in this study; therefore, the evaluation of zinc chloride mouthwash on the incidence and severity of mucositis in other mucous membranes of the gastrointestinal tract could yield findings that are more valuable.

## Conclusion

Zinc chloride mouthwash in cancer patients undergoing chemotherapy has properly promoted oral health and reduced the incidence and severity of oral mucositis in these patients from the start of chemotherapy until about three weeks later. Thus, Caregivers, especially nurses in chemotherapy wards can recommend zinc chloride as a therapeutic care measure for these patients to improve oral health and subsequently reduce the pain and suffering caused by the side effects of chemotherapy.

## Data Availability

The datasets used and/or analyzed during the current study are available from the corresponding author on reasonable request and subject to compliance with patient privacy regulations.
